# Cold water swimming as an add-on treatment for depression: a feasibility study

**DOI:** 10.1192/j.eurpsy.2024.1109

**Published:** 2024-08-27

**Authors:** P. Hjorth, M. R. V. Rasmussen

**Affiliations:** ^1^Psychiatry, Psychiatric Department, Vejle. Mental Health Services, Region of Southern Denmark, University Hospital of Southern Denmark. Institute of Regional Health research, University of Southern Denmark, Odense, Denmark; ^2^Psychiatry, Vejle Psychiatric hospital, Vejle, Denmark

## Abstract

**Introduction:**

In Denmark, 14% of patients with depression develops treatment resistant depression (TRD) after the first hospital contact. Explanations for TRD include lack of clinical effect of pharmacological treatment and reluctance to treatment due to price, discomfort, and unacceptable side effects. Cold water swimming (CWS) describes swimming outdoors during the winter season in cold to ice-cold water on a regular basis. Many winter swimmers believe that exposure to cold water is beneficial for their health. However, evidence of health effects have been anecdotal or based on results from small sample-size studies. The availably studies report that winter swimming abolishes general tiredness, boosts self-esteem and improves mood and/or general well-being.

**Objectives:**

Aims To test if it is possible for patients with depression to participate in two weekly sessions of CWS and to measure the effects of CWS on general well-being and depression.

**Methods:**

All psychiatric in- and outpatients from the department of psychiatry at Little Belt Hospital, Vejle with a diagnose of depression were eligible for inclusion. CWS-sessions included a dip in an inlet - and a short swim for a few minutes – depending on individual preferences.

**Results:**

The average water temperature was 7.5 grades C. The lowest water temperature was 2.0 grades C. 13 patients were participating in CWS sessions. One of the patients participated in 40 CWS sessions and the average number of CWS session was 14.5 (sd: 11.2). The participating patients were on average overweight, and they had mild to severe sleep problems with an average score of 10.1 (sd: 3.7) on Pittsburg Sleep Quality index. Patients with regular CWS have a wellscore of 39.2 and at the end of the swimming season, their score has increased to 54.0. Sleep: At index for regular swimmers, the score was 10.4 and at the end of season in had decreased to 8.0 while the patients’ not regular swimming had an unchanged score of 11.3. After each CWS sessions, a cheerful and uplifted atmosphere spread among the participants and the conversation afterwards was often characterized by this.

**Conclusions:**

The nurses had an important task and function in guidance to the participating patients due to the patients’ symptoms from depression. It was surprisingly easy to get all the patients to swim in the cold water. Due to the design and small numbers of participants in this feasibility study, it is not possible to draw any statistically significant results. Nevertheless, we can conclude that it is possible to use CWS as a treatment opportunity for some patients with depression. The research group members are convinced that for some patients it will be an important part of recovery from depression. Further studies with control group and a statistical satisfying larger group of participants will probably generate more knowledge’s on these issues.

**Disclosure of Interest:**

None Declared

